# Social Class, Social Mobility and Risk of Psychiatric Disorder - A Population-Based Longitudinal Study

**DOI:** 10.1371/journal.pone.0077975

**Published:** 2013-11-15

**Authors:** Sanna Tiikkaja, Sven Sandin, Ninoa Malki, Bitte Modin, Pär Sparén, Christina M. Hultman

**Affiliations:** 1 Department of Medical Epidemiology and Biostatistics, Stockholm, Sweden; 2 Centre for Health Equity Studies (CHESS), Stockholm University, Stockholm, Sweden; University of Florida, United States of America

## Abstract

**Objectives:**

This study explored how adult social class and social mobility between parental and own adult social class is related to psychiatric disorder.

**Material and Methods:**

In this prospective cohort study, over 1 million employed Swedes born in 1949-1959 were included. Information on parental class (1960) and own mid-life social class (1980 and 1990) was retrieved from the censuses and categorised as High Non-manual, Low Non-manual, High Manual, Low Manual and Self-employed. After identifying adult class, individuals were followed for psychiatric disorder by first admission of schizophrenia, alcoholism and drug dependency, affective psychosis and neurosis or personality disorder (N=24 659) from the Swedish Patient Register. We used Poisson regression analysis to estimate first admission rates of psychiatric disorder per 100 000 person-years and relative risks (RR) by adult social class (treated as a time-varying covariate). The RRs of psychiatric disorder among the Non-manual and Manual classes were also estimated by magnitude of social mobility.

**Results:**

The rate of psychiatric disorder was significantly higher among individuals belonging to the Low manual class as compared with the High Non-manual class. Compared to High Non-manual class, the risk for psychiatric disorder ranged from 2.07 (Low Manual class) to 1.38 (Low Non-manual class). Parental class had a minor impact on these estimates. Among the Non-manual and Manual classes, downward mobility was associated with increased risk and upward mobility with decreased risk of psychiatric disorder. In addition, downward mobility was inversely associated with the magnitude of social mobility, independent of parental class.

**Conclusions:**

Independently of parental social class, the risk of psychiatric disorder increases with increased downward social mobility and decreases with increased upward mobility.

## Introduction

Social class differences in health have been documented throughout the Western world for most major health and mortality outcomes [1-4]. There are several possible factors that may explain the social class differences in health, such as lifestyle, work-related stress, working conditions and financial strain [1,2]. Psychiatric disorder, present in about 10% of the adult population at some point across the life-course, is a major global public health problem [5]. Previous studies have shown an inverse relation between adult social class and risk for psychiatric disorder [6,7]. In a similar manner, poor psychiatric health in adult life has been linked to low parental social class [8,9].

Social mobility, from parental to adult social class, is suggested to be an important aspect of social stratification that may affect the development of psychiatric disorder [9,10]. Social mobility patterns of psychiatric patients has been studied since the 1950’s [9,11-13], whereas the reverse association between social mobility and subsequent psychiatric disorder has been less explored [10,14,15]. Downward mobility has been linked to an increased risk of poor self-reported mental health [14] and alcoholism [10] among men, but not women. The focus of this paper is to study how social class and social mobility is related to the risk of subsequent psychiatric disorder. 

### Aims

Our aim was to examine whether adult social class and social mobility between parental and own adult social class is related to subsequent psychiatric disorder.

## Materials and Methods

We utilised information from several Swedish population-based, nation-wide registries in this study. All Swedish residents are assigned a unique identification number that stays the same throughout life. This identification number enables linkage of individuals between nationwide Swedish registers by e.g. Statistics Sweden and the National Board of Health and Welfare. Before giving out data for research, the original identification numbers were replaced by a sequence number, unique to each individual. Information about social class was retrieved from the Swedish censuses in 1980 and 1990 and information about parental social class was retrieved from the 1960 Census. The Census-questionnaires were sent to all Swedish registered residents, with mandatory response. The response rate was 99% in the 1960 and 1980 [16] and 98% in the 1990 Census [17]. The censuses included detailed questions about occupations and socioeconomic indicators that allowed construction of the Swedish socioeconomic index (SEI), which was used for classifying social class [18]. The SEI categories are similar to the Erikson, Goldthorpe and Portocarrero (EGP) social class scheme (Table S1). The Swedish Multi-Generation Register, which includes individuals born 1932 or later who lived in Sweden 1961 or later, was used to link children with their parents [19]. The Swedish Patient Register provided data on hospital discharges and diagnoses classified according to the World Health Organisation’s International Classification of Diseases (ICD). The register has a nationwide coverage of patient treatment facilities and includes care in psychiatric as well as somatic hospitals. Sweden has universal and publicly financed health insurance coverage that guarantees equal access to health services, regardless of employment status, socio-economic status or place of residency. The register contains diagnostic information by the treating physician, date of admission and discharge and the name of hospital on virtually all psychiatric hospitalisations since 1973 [20]. The diagnoses are most often given by a specialist in psychiatry and based on observations made during hospitalisation, evaluation of the service user and medical records at discharge. The diagnostic assessment is then forwarded electronically in standardised manner to the National Patient Register. The diagnoses used here, from the register of hospitalised patients, capture severe psychiatric conditions. We used ICD versions 8-10 to identify psychiatric patients and ICD versions 7-9 to identify parental psychiatric disorder (Table S2). The ICD discharge diagnoses for psychiatric disorders recorded in the Swedish Registers are in agreement with diagnoses based on Diagnostic and Statistical Manual of Mental Disorders (DSM) criteria and with those based on semi-structured interviews and medical records [21-23]. The Causes of Death [24] and Total Population Register [25] were used to censor individuals due to death or emigration. 

### Materials

**Figure 1:**
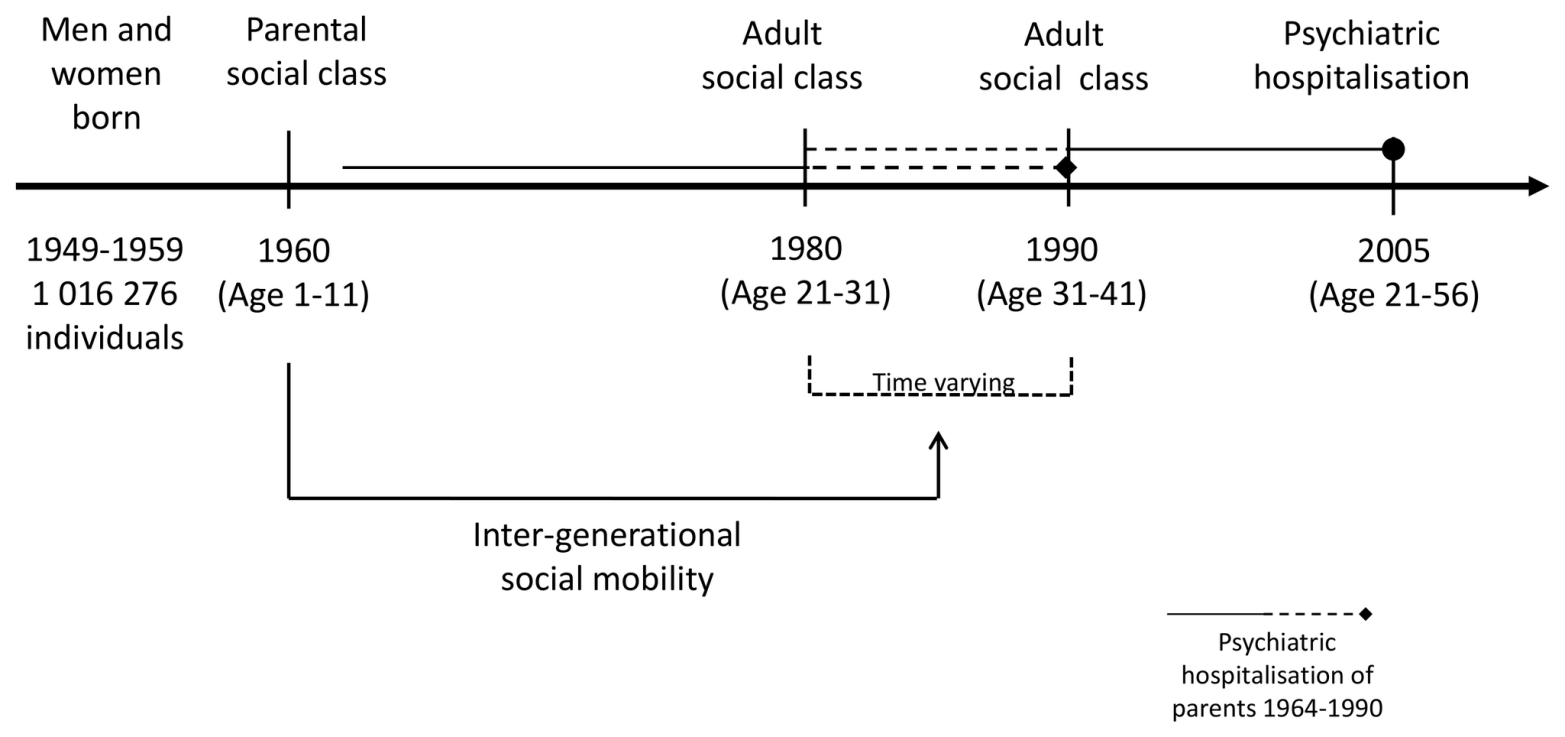
Overview of the time-points at which information for the studied subjects and their parents has been retrieved.

In this prospective cohort study, we included all Swedish individuals born in 1949–1959 who, according to both parental class in 1960 and own adult class in 1980 or 1990, could be linked to an occupational class (Manual, Non-manual, or Self-employed) (Figure 1). We defined psychiatric patients as individuals with a first psychiatric inpatient admission during the period 1980–2005. This group included patients with schizophrenia (N=477), alcoholism (N=11 323) and drug dependency (N=1 850), affective psychosis (N=1 440) and neurosis and personality disorder (N=9 569). Five social classes were used to categorise adult and parental (1960) and adult social class (1980 or 1990): High Non-manual (including intermediate Non-manual), Low Non-manual, High Manual, Low Manual and Self-employed (including farmers). Parental social class was based on the head of the household [26] while adult class was based on the individual’s own class. A conceptual model of social class, social mobility in relation to psychiatric disorder is displayed in Figure S1. For the Non-manual and Manual classes, trajectory-specific social mobility was analysed as downward (-1, -2, -3 steps), upward (+1, +2, +3 steps) and stable (no mobility). The Self-employed were excluded in this part of the analyses due to the difficulty to place this group into a hierarchical order. 

We excluded individuals born outside Sweden (N= 14 113), because of differences in both social class attainment and occurrence of psychiatric disorders [27]. Immigrants may be subjected to other as well as additional obstacles to enter the labour market. Further, they may have other types of psychological trauma (from their country of origin) that lead to disparities in psychiatric diagnosis [28]. Individuals who did not have information on adult (N=64 584) or parental (N=43 912) social class were also excluded. Psychiatric disorder was present among 8% of those lacking adult social class information and 5% among those lacking parental social class information, whereas the corresponding figures for included subjects were 2% in both cases. Sex and age were similarly distributed in missing versus non-missing data.

To get a more accurate measurement of the effects of social class on subsequent psychiatric disorder, individuals with a psychiatric admission prior to the measurement of adult class were also excluded (N=18 126). In all, 1 016 276 individuals were included in this study, of which 24 659 individuals were hospitalised due to psychiatric disorder during follow-up. Any parental psychiatric hospitalisation occurring before the measurement of adult class in 1980/1990 was identified and grouped according to whether the mother, the father, or both parents had been hospitalised for such a condition. Additional variables included were birth cohort (1949–1954, 1955–1959), age at diagnosis (21–25, 26–30, 31–35, 36–40, 41–45, 46–50, 51–56) and sex.

### Statistical Methods

To analyse the probability of psychiatric disorder as a function of adult and parental social class, we fitted Poisson regression models adjusting for sex and age at diagnosis. We followed each subject from the first measurement of adult class (1980 or 1990) until date of first (incident) psychiatric admission, death, emigration or December 31, 2005, whichever came first. In Poisson regression models we estimated the rate (cases per 100 000 person- years) of psychiatric disorder, by adult social class and time intervals of age at diagnosis as well as relative risks (RR) with two-sided 95% Wald type confidence intervals. In a second step we adjusted for parental social class and parental psychiatric history. The analysis allowed for differences in length of follow-up and the subjects to change social class during follow-up. In an additional analysis, we included an interaction term between parental and adult social class and then calculated the RR of psychiatric disorder associated with different trajectories of social mobility for a graphic presentation. The RRs for psychiatric disorder by adult class and social mobility trajectories were also calculated separately for men and women (shown in Table S3, Figure S2 and Figure S3). Ethical approval was obtained from the Regional Ethical Review Board, Karolinska Institutet, Sweden.

## Results

Psychiatric disorder was more common among men (N=15 253) than women (N=9 406) (Table 1). Individuals with parental psychiatric disorder or low parental social class had higher rates of psychiatric disorder than individuals without parental psychiatric history or high parental social class.

**Table 1:** Background characteristics of psychiatric patients.

**Sex**	Number of Subjects (rate*)
Men	15 253 (123)
Women	9 406 (86)
**Birth cohort**	
1949-1954	13 525 (103)
1955-1959	11 134 (108)
**Parental psychiatric disorder**	
Father	2 173 (170)
Mother	1 614 (164)
Both parents	324 (284)
No parental psychiatric history	20 548 (98)
**Parental class**	
High Non-manual	4 090 (89)
Low Non-manual	2 587 (108)
High Manual	6 643 (116)
Low Manual	7 312 (120)
Self -employed	4 027 (87)

* Rate of psychiatric disorder per 100 000 person-years.

(N=24 659) and rates for psychiatric disorder.

**Figure 2:**
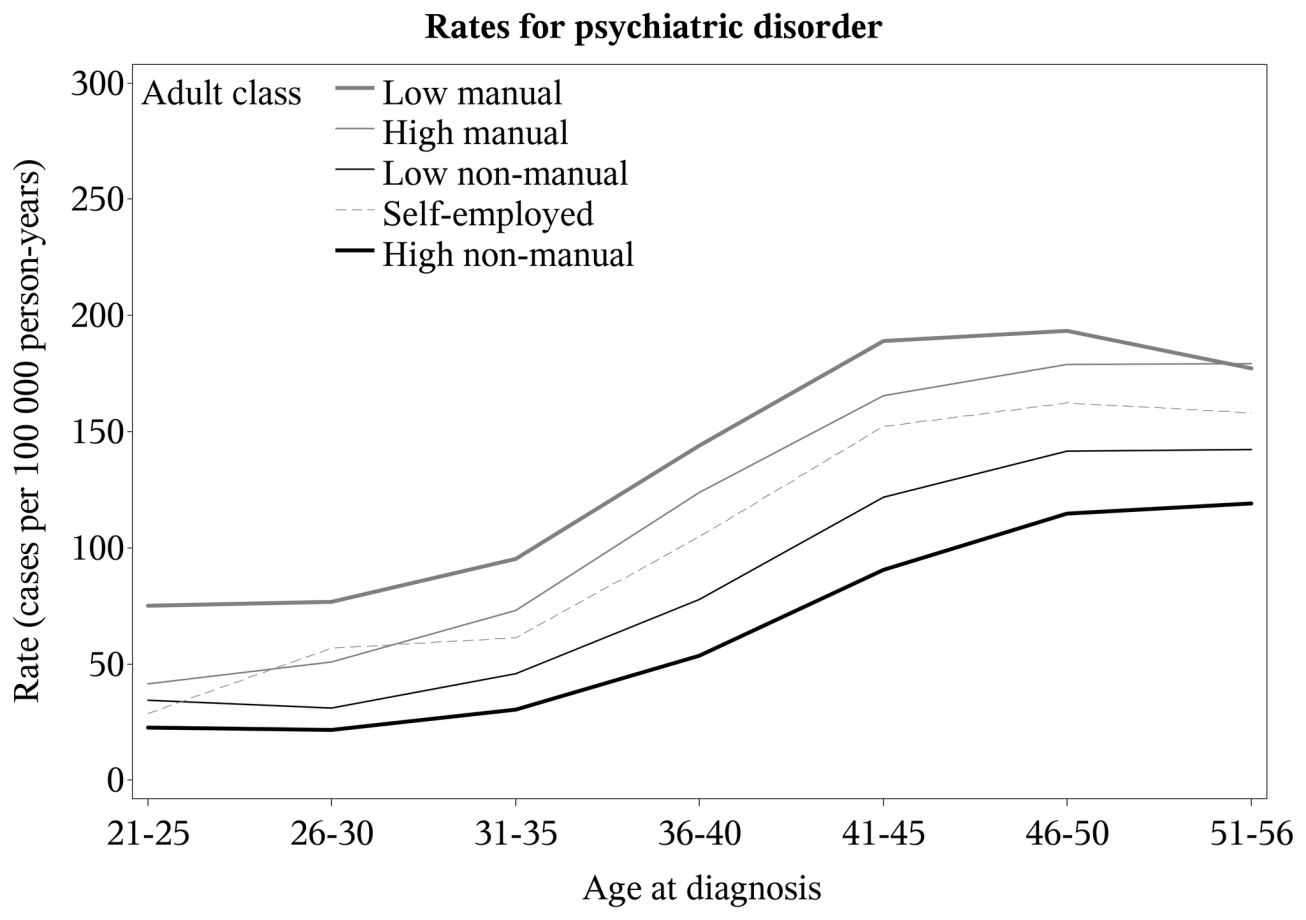
Rate of psychiatric disorder (cases per 100 000 subjects) versus age at diagnosis. Rate developments by adult social class at cohort entry. Footnote: Subjects=1 016 276; psychiatric patients: 24 659. Swedish born in 1949–1959.

The rates of psychiatric disorder varied by adult social class and increased in an approximately parallel fashion between classes with age at diagnosis (Figure 2). At ages 41-45 years, the rates were 90 (95% CI: 86-95) for High Non-manual class, 121 (95% CI: 114-129) for Low Non-manual class, 165 (95% CI: 157-174) for High Manual class, 189 (95% CI: 181-197) for Low Manual class, and 152 (95% CI: 139-166) for Self-employed. 

The RRs for psychiatric disorder varied by adult social class (Table 2, Model 1), with higher risks for the Manual and Self-employed classes than for the Non-manual classes. The RR estimates decreased slightly after additional adjustment for parental class and parental psychiatric disorder, except for the Self-employed (Table 2, Model 2). This finding indicates that parental social class is not a confounder in the relation between adult social class and psychiatric disorder. In an additional model including the Non-manual and Manual classes (N= 798 660, psychiatric patients N= 19 553), we estimated the interaction term between parental and adult class. The estimate for the interaction term was not statistically significant (p=0.17) (Table 2). Further, in the sex-specific analysis the risks for psychiatric disorder followed the same pattern, although the RRs were consistently higher among men than women (Table S3). The interaction term estimate of social mobility was significant for men (p=0.03) but not for women (p=0.68).

**Table 2:** Relative risk (RR) of psychiatric disorder and two-sided 95% confidence intervals (CI) comparing subjects with different adult social class.

	**Model 1**	**Model 2**
**Adult social class**	**RR (CI)**	**RR (CI)**
High Non-manual	Reference category	Reference category
Low Non-manual	1.38 (1.32-1.44)	1.34 (1.29-1.40)
High Manual	1.66 (1.60-1.73)	1.60 (1.54-1.67)
Low Manual	2.07 (2.00-2.14)	1.98 (1.91-2.05)
Self-employed	1.53 (1.45-1.61)	1.55 (1.47-1.64)
Social mobility*	p=0.17	

Model 1 Adjusted for Sex, Age at Diagnosis (21–25, 26–30, 31–35, 36–40, 41–45, 46–50, 51–56) and Birth Cohort (1949–1954, 1955–1959)

Model 2 adjusted for sex, age at diagnosis (21–25, 26–30, 31–35, 36–40, 41–45, 46–50, 51–56), birth cohort (1949–1954, 1955–1959), parental social class (High Non-manual, Low Non-manual, High Manual, Low Manual, Self-employed) and parental psychiatric disorder (father, mother, both parents, no parental psychiatric history).

* Statistical test of interaction between parental social class and adult social class among the Non-manual and Manual classes.

**Figure 3:**
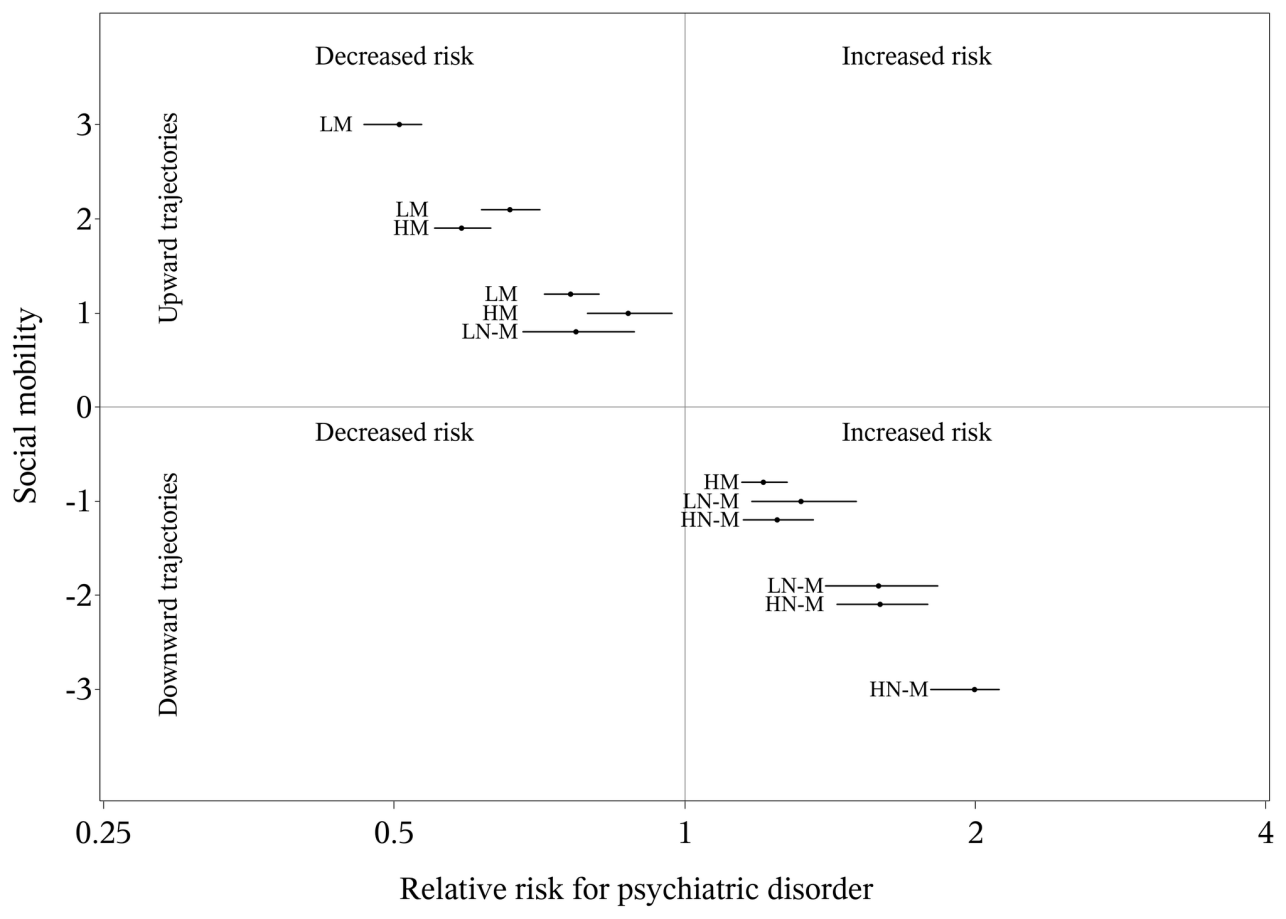
Relative risk (x-axis) of psychiatric disorder and two-sided 95% confidence intervals comparing different trajectories of social mobility (y-axis -3 to +3) versus subjects socially stable (stable between parent class to adult class; reference group). For each trajectory (-3, -2 ,..., +3) different relative risks presented for different parental class. Footnote: Trajectories start from high non-manual (HN-M) parental class, low non-manual (LN-M) parental class, high manual (HM) parental class, low manual (LM) parental class by upward (y-axis +1, +2 or +3) and downward mobility ( y-axis -3, -2, -1) with their corresponding RRs and 95% CIs. Subjects=798 660; psychiatric patients:19 533.

Analysis of magnitude of social mobility among the Manual and Non-manual classes showed that all upward trajectories had statistically significantly lower risks, whereas all downward trajectories had statistically significantly higher risks, compared to those remaining stable in the respective class (Figure 3). The larger the movement, the greater was the change in risk. Comparing the risks of individuals who moved equivalent distances (-2, -1, +1, +2 steps), but from different starting points, revealed similar estimates with overlapping CI:s, independent of parental social class.

The magnitude of social mobility patterns was more pronounced for men than for women (Figure S2 and Figure S3); otherwise it followed the same pattern, with a few exceptions. The lower risks of psychiatric disorder among upwardly mobile was not statistically significant for all sex specific trajectories (+1 step). Men who moved upward 2 steps from High Manual class had a lower risk of psychiatric disorder than men moving the same distance, but from Low Manual class. This was not the case for women, however. Additional analyses were also carried out separately for men and women using the diagnostic groups of alcohol or neurosis and personality disorder as outcome. The results were similar as presented in Figure S2 and S3 (data not shown). 

## Discussion

### This study includes over one million individuals who were employed in Sweden during

1980/1990, for whom subsequent psychiatric disorder was followed-up from 1980-2005 (24 656 psychiatric patients). We assessed psychiatric disorder according to adult class and intergenerational social mobility. We found that the risk of psychiatric disorder differed in relation to adult class and that this association could not be explained by parental class. The risk of psychiatric disorder was considerably higher among the lower than among the higher social classes. Overall, upward mobility was associated with a lower risk of psychiatric disorder, in contrast downward mobility was associated with a higher risk, irrespective of parental social class. However, socially mobile individuals, who experienced similar magnitude of social mobility, displayed similar increase or decrease in risk of psychiatric disorder, compared to those who were stable.

### Our findings in relation to previous studies

Our findings of the importance of adult class, rather than parental class, on psychiatric disorders, are confirmed by previous studies among men [9,10,14] and in a joint analysis of men and women [29]. The disparities in rates of psychiatric disorder between adult social classes are also consistent with a previous study from Sweden [9]. Individuals in lower social classes often face difficulties, such as poor economic, environmental or working conditions, which may lead to higher prevalence of psychiatric disorder, compared to those in higher social classes [30].

In line with our findings, previous studies have shown that downward social mobility [10,14,15 ] and downward educational mobility [31] are associated with alcoholism [10], poor self-reported mental health [14] and depressive symptoms [15]. However, no study has assessed the effect of social mobility on the risk of psychiatric disorder as an interaction between parental and adult class, therefore not being able to separate the main effects of parental and adult social class from social mobility per se. Moreover, the lower risk for psychiatric disorder among the upwardly mobile, has not been shown previously, nor has the consistent relationship between magnitude of social mobility and risk of psychiatric disorder. We can only speculate about the reasons for these findings. Resources connected to low social class across the life course may induce chronic stress, which, in turn may trigger the development of psychiatric disorder [32]. A low level of education or poor control over working-life (low job control or insecure employment) may result in destructive ways of handling the stressors accompanied by such conditions, such as alcohol or drug abuse [32]. Socially mobile individuals may change their health behaviours [33,34] as they adjust their manners and lifestyle habits to “live up to” (or “live down to”) to their social class of destination [35]. It has also been suggested that socially mobile individuals experience stress, because of the social class change itself [36], with downward mobility being more disruptive than upward mobility [35]. This view is supported by the present findings, since the downwardly mobile display an increased risk of psychiatric disorder. The upwardly mobile may also have coping skills to handle these stressors (e.g. because of a higher educational level) [32] which may lower the risks of psychiatric disorder. Previous findings have shown that upwardly mobile individuals have similar stress levels as the socially stable [37].

Our results also indicated that the distance moved is associated with the risk of psychiatric disorder. Regardless of social mobility trajectory, individuals who experienced similar magnitude of social mobility had similar risks for psychiatric disorder. Furthermore, the effects of social mobility, measured as the interaction between parental and adult class, was significantly associated with psychiatric disorder among men, but not among women. On the other hand, if the psychiatric patients in our study had symptoms previous to their hospitalisation, several inter-related factors may have influenced our results: patients may have been *health selected* into a (low) social position and the disorder may have therefore *caused* the downward social mobility. 

In this study, we tried to account for genetic and other influences by adjusting for parental psychiatric disorder [29,38,39]. However, adjusting for parental psychiatric history had only a minor effect on the association between adult class, social mobility and risk of psychiatric disorder. We chose to measure a broad spectrum of parental psychiatric history and therefore we used any parental psychiatric disorder occurring before measurement of adult social class (1980/1990) and did not make any diagnosis-specific adjustments. It is possible that we did not capture the true effect of parental psychiatric history for adult psychiatric disorder, because parents may have been hospitalised earlier than 1964 (not covered by our data) or during the follow-up period of our study subjects, starting from 1980 (Figure 1).

### Strengths and Limitations

We utilised extensive detailed measurements of social class, with follow-up of covariates over ten years. Our data allowed us to divide the Non-manual and Manual classes into High and Low, which is seldom possible in the analysis of social mobility between generations. We also included the category Self-employed when analysing the effects of adult and parental class on the risk of psychiatric disorder. The Self-employed were a large group in 1960 [40], but has, decreased in size through 1990 because of structural changes in society [41]. Because of difficulties in placing the Self-employed into the social hierarchy, they were excluded from the trajectory specific social mobility analysis. One strength of this study is that both men and women were included and analysed separately, while previous studies focused solely on men [10] or did not carry out separate analyses for men and women [15]. We found that an effect of social mobility *per se* on risk of psychiatric disorder was significant among men, but not among women. The lack of effect among women may also be a power issue, since the majority of the psychiatric patients were men. One limitation is that we lack information on outpatient care (available from 2001). Cases with milder forms of psychiatric problems who were treated only as outpatients would have been classified as unaffected, which probably leads to an underestimation of the association between social class and psychiatric disorder. Sweden experienced a psychiatric care reform in the 1990’s, leading to downsizing of psychiatric hospital care and opening of outpatient care clinics. As a result, the number of inpatient hospitalisations has decreased during the course of our study, whereas outpatient care has increased [20,42]. To our advantage, the diagnostic data used on the included patients is of very high quality [20]. 

Because the focus of this paper was to study the effects of social class and social mobility, only individuals who were employed in 1980/1990 were included. The participants were included in the analyses only if they were free of psychiatric admission before the measurement of adult social class. This strategy was necessary to minimise the effect of reversed causation. With this design, we were able to examine the role of social class and social mobility for later psychiatric disease in a more careful way than previous studies have managed to do. Our results confirm that there is an association between social class, social mobility and psychiatric disorder. 

From the clinical perspective, low social class and unstable working conditions are among risk factors to consider for psychiatric disorders. Even if the present study does not focus on implications on the practical level, it highlights the importance of continuous effort to facilitate for individuals to maintain a connection to the labour market.

## Conclusions

The risk of psychiatric disorder is inversely related to social class. Independently of parental social class, the risk of psychiatric disorder increases with increased downward social mobility and decreases with increased upward mobility.

## Supporting Information

Figure S1 **A conceptual model of social class, social mobility and psychiatric disorder.**
(TIF)

Figure S2 **Relative risk (x-axis) of psychiatric disorder among men and two-sided 95% confidence intervals comparing different trajectories of social mobility (y-axis -3 to +3) versus subjects socially stable (stable between parent class to adult class; reference group).** For each trajectory (-3, -2 ,..., +3) different relative risks presented for different parental class.
Footnote Figure S2: Trajectories start from high non-manual (HN-M) parental class, low non-manual (LN-M) parental class, high manual (HM) parental class, low manual (LM) parental class by upward(y-axis +1, +2 or +3) and downward mobility (y-axis -3, -2, -1) with their corresponding RRs and 95% CIs. Subjects=412 462; psychiatric patients:11 889.
(TIF)

Figure S3 **Relative risk (x-axis) of psychiatric disorder among women and two-sided 95%: confidence intervals comparing different trajectories of social mobility (y-axis -3 to +3) versus subjects socially stable (stable between parent class to adult class; reference group).** For each trajectory (-3, -2 ,..., +3) different relative risks presented for different parental class.
Footnote Figure S3: Trajectories start from high non-manual (HN-M) parental class, low non-manual (LN-M) parental class, high manual (HM) parental class, low manual (LM) parental class by upward(y-axis +1, +2 or +3) and downward mobility ( y-axis -3, -2, -1) with their corresponding RRs and 95% CIs. Subjects=386 198; psychiatric patients:7 664.
(TIF)

Table S1 **The Swedish socio-economic classification (SEI).**
(TIF)

Table S2 **ICD codes used for identification of psychiatric disorder.**
(DOCX)

Table S3 **Sex specific relative risk (RR) of psychiatric disorder and two-sided 95% confidence intervals (CI) comparing subjects with different adult social class.** Footnote Table S3: Subjects= Men 527 384 psychiatric patients; 15 253, Women 488 892; psychiatric patients 9 406. Swedish born 1949=1959.Models for men and women were fitted separately.
Model 1 adjusted for age at diagnosis (21–25, 26–30, 31–35, 36–40, 41–45, 46–50, 51–56) and birth cohort (1949–1954, 1955–1959).
Model 2 adjusted for age at diagnosis (21–25, 26–30, 31–35, 36–40, 41–45, 46–50, 51–56) birth cohort (1949–1954, 1955–1959), parental social class (High Non-manual, Low Non-manual, High manual, Low manual, Self-employed) and parental psychiatric disorder (father, mother, both parents, no parental psychiatric history).
* Statistical test of interaction between parental social class and adult social class among the Non-manual and Manual classes.
(DOCX)
