# Supplementary figures and images for: Social Class, Social Mobility and Risk of Psychiatric Disorder - A Population-Based Longitudinal Study
Source: PLoS One. 2013 Nov 15;8(11):e77975. doi: 10.1371/journal.pone.0077975 (PMC3829839; doi:10.1371/journal.pone.0077975)

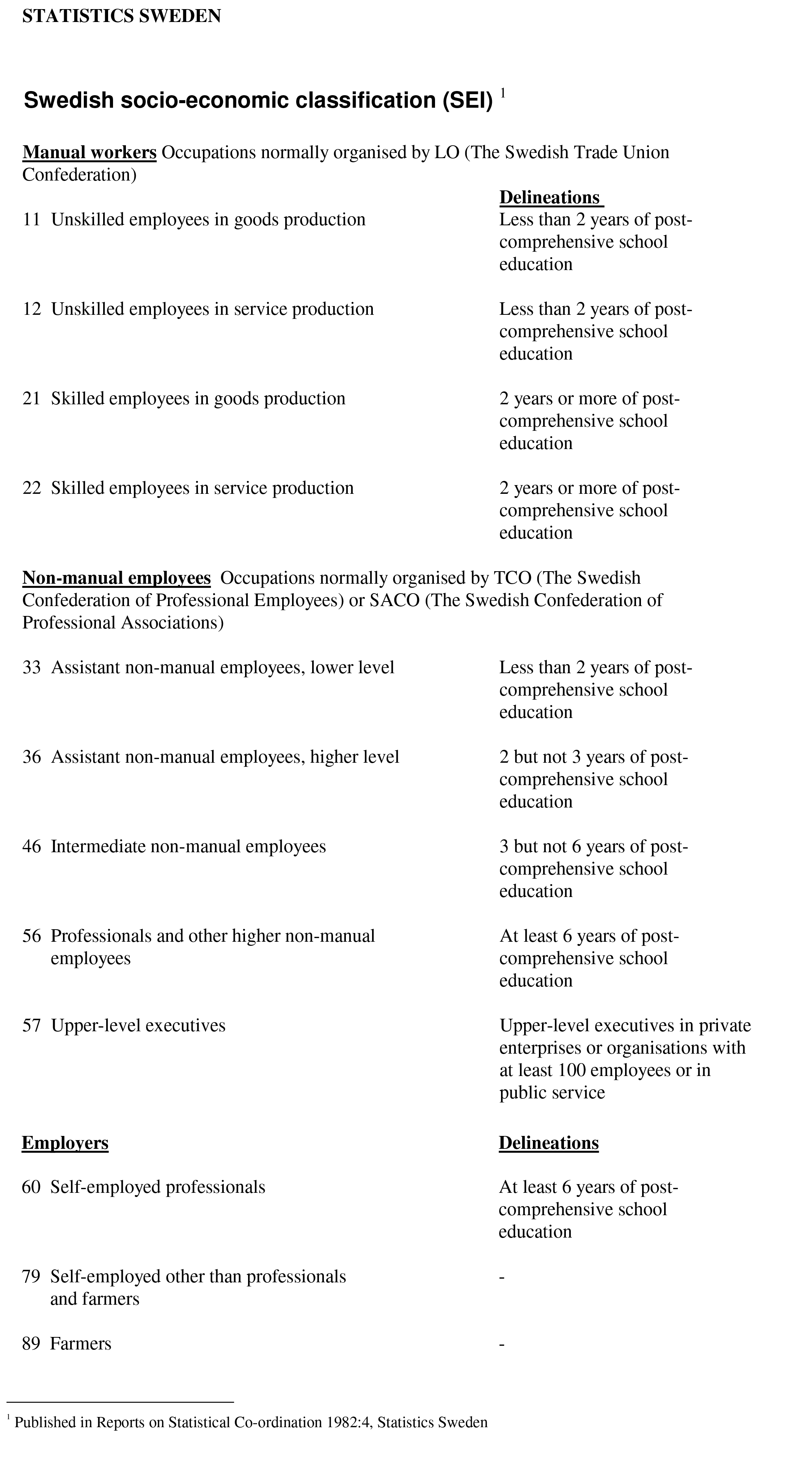

Supplement: Table S1 — The Swedish socio-economic classification (SEI). (TIF) [file pone.0077975.s004.tif]
