# Supplementary material for: Social Class, Social Mobility and Risk of Psychiatric Disorder - A Population-Based Longitudinal Study
Source: PLoS One. 2013 Nov 15;8(11):e77975. doi: 10.1371/journal.pone.0077975 (PMC3829839; doi:10.1371/journal.pone.0077975)
Supplement: Table S2 — ICD codes used for identification of psychiatric disorder. (DOCX) [file pone.0077975.s005.docx]

| **Individual**  **diagnosis** | **ICD-8 (1969-1986)** | **ICD-9**  **(1987-1996)** | **ICD-10 (1997-2005)** |
| --- | --- | --- | --- |
| Schizophrenia | 295 | 295 | F20 |
| Alcoholism and drug dependency | 303,304 | 291, 303, 305A, 292, 304,305X | F10, F19 |
| Affective psychosis | 296 | 296 | F30,F31 |
| Neurosis and personality disorder | 300-301 | 300-301 | F32- F34, F41- F43 |
| **Parental psychiatric**  **diagnosis** | **ICD-7**  **(1964-1968)** | **ICD-8**  **(1969-1986)** | **ICD-9**  **(1987-1996)** |
| Any psychiatric disorder | 300-326 | 290-315 | 290-319 |

ICD: World Health Organisation’s International Classification of Diseases.
