# Supplementary material for: Social Class, Social Mobility and Risk of Psychiatric Disorder - A Population-Based Longitudinal Study
Source: PLoS One. 2013 Nov 15;8(11):e77975. doi: 10.1371/journal.pone.0077975 (PMC3829839; doi:10.1371/journal.pone.0077975)
Supplement: Table S3 — Sex specific relative risk (RR) of psychiatric disorder and two-sided 95% confidence intervals (CI) comparing subjects with different adult social class. Footnote Table S3: Subjects= Men 527 384 psychiatric patients; 15 253, Women 488 892; psychiatric patients 9 406. Swedish born 1949=1959.Models for men and women were fitted separately. Model 1 adjusted for age at diagnosis (21–25, 26–30, 31–35, 36–40, 41–45, 46–50, 51–56) and birth cohort (1949–1954, 1955–1959). Model 2 adjusted for age at diagnosis (21–25, 26–30, 31–35, 36–40, 41–45, 46–50, 51–56) birth cohort (1949–1954, 1955–1959), parental social class (High Non-manual, Low Non-manual, High manual, Low manual, Self-employed) and parental psychiatric disorder (father, mother, both parents, no parental psychiatric history). * Statistical test of interaction between parental social class and adult social class among the Non-manual and Manual classes. (DOCX) [file pone.0077975.s006.docx]

| **Adult class** | **Men Model 1** | **Men Model 2** | **Women Model 1** | **Women Model 2** |
| --- | --- | --- | --- | --- |
| High Non-manual | Reference category | Reference category | Reference category | Reference category |
| Low Non-manual | 1.51 (1.42-1.60) | 1.47 (1.38-1.57) | 1.22 (1.15-1.30) | 1.20 (1.12-1.27) |
| High Manual | 1.80 (1.71-1.88) | 1.72 (1.64-1.81) | 1.44 (1.34-1.55) | 1.40 (1.31-1.51) |
| Low Manual | 2.29 (2.18-2.39) | 2.19 (2.08-2.29) | 1.78 (1.69-1.88) | 1.72 (1.62-1.81) |
| Self-employed | 1.64 (1.53-1.74) | 1.69 (1.58-1.81) | 1.31 (1.17-1.47) | 1.32 (1.18-1.48) |
| Social mobility* | p=0.03 |  | p=0.69 |  |
